# Editorial: Hepatocellular carcinoma: from bench to bedside

**DOI:** 10.3389/fgene.2026.1901215

**Published:** 2026-07-07

**Authors:** Evin İşcan, Aaron B. Koenig, Xiaogang Wu

**Affiliations:** 1 Izmir Biomedicine and Genome Center (IBG), Dokuz Eylul University Health Campus, Izmir, Türkiye; 2 Izmir International Biomedicine and Genome Institute (IBG-Izmir), Dokuz Eylul University, Izmir, Türkiye; 3 Celia Scott Weatherhead School of Public Health and Tropical Medicine, Tulane University, New Orleans, LA, United States; 4 The Global NASH/MASH Council, Washington, DC, United States; 5 The University of Texas MD Anderson Cancer Center, Houston, TX, United States

**Keywords:** HCC immune landscape, HCC prognosis, HCC screening, HCC therapy, hepatitis B-related hepatocellular carcinoma, hepatocellular carcinoma, neoadjuvant chemotherapy, tumor microenvironment-TME

Hepatocellular carcinoma (HCC) represents the predominant form of primary liver cancer, accounting for nearly 90% of all liver malignancies (Ch et al., 2021). Despite considerable advances in diagnostic and therapeutic approaches, HCC continues to impose a substantial global health burden, with its incidence and mortality rates steadily increasing worldwide (Llovet et al., 2021; Sia et al., 2017). Although potentially curative interventions, including surgical resection, liver transplantation, and local ablative therapies, are available for patients diagnosed at an early stage, the majority of cases are detected at more advanced stages, when treatment options become limited and clinical outcomes remain poor. HCC exhibits profound molecular heterogeneity driven by a broad spectrum of genetic and epigenetic alterations. Consequently, elucidating the molecular events that govern HCC development and progression remains critical for the discovery of novel biomarkers and the development of more effective therapeutic strategies. In this Research Topic (RT), comprising 12 articles, we aim to bring together original research and review articles that provide new insights into the molecular mechanisms, clinical characteristics, and emerging therapeutic strategies associated with HCC. For this RT, we have received several papers that address important clinical and biological aspects of HCC, ranging from therapeutic strategies and prognostic tools to tumor microenvironment dynamics and molecular biomarkers.

Several contributions focused on improving therapeutic approaches for HCC. In their original article, Zhang et al. compared transarterial chemoembolization combined with donafenib (TACE + D) and transarterial chemoembolization combined with donafenib and camrelizumab (TACE + D + C) in patients with unresectable HCC. The authors demonstrated that the addition of camrelizumab significantly improved treatment responses and survival outcomes while maintaining a manageable safety profile. Similarly, Xue et al. evaluated the efficacy of transarterial chemoembolization combined with lenvatinib and PD-1 blockade in patients with intermediate-stage HCC exceeding the up-to-7 criteria. Their findings showed improved progression-free survival, particularly in progression to macrovascular invasion or extrahepatic spread, further supporting the growing role of immunotherapy-based combination strategies in HCC treatment. Complementing these studies, Wu et al. performed a systematic review and meta-analysis evaluating neoadjuvant systemic therapies in resectable HCC. Their results demonstrated encouraging pathological and clinical response rates, particularly for treatment regimens combining targeted agents and immune checkpoint inhibitors (Wu et al.).

While these studies focused on therapeutic interventions, others addressed factors influencing surgical outcomes and clinical management. In this regard, Gao et al. investigated the impact of portal hypertension on surgical outcomes in patients undergoing hepatectomy. Although portal hypertension was associated with less favorable perioperative outcomes, long-term survival outcomes remained largely comparable between patient groups, highlighting its complex role in patient selection and surgical decision-making. Several studies also addressed the growing need for more accurate diagnostic and prognostic tools in HCC. Ye et al. developed and validated a prognostic nomogram for recurrence-free survival in patients with microvascular invasion-negative HCC. By integrating clinicopathological and inflammatory parameters, the model demonstrated strong predictive performance and may facilitate postoperative risk stratification. Likewise, Zhang et al. developed a non-invasive nomogram capable of differentiating alpha-fetoprotein-negative HCC from other intrahepatic malignant lesions. By combining contrast-enhanced ultrasound findings with clinical characteristics, the model achieved high diagnostic accuracy and may improve individualized decision-making during HCC screening. In a related study, Wu et al. evaluated the prognostic value of C-reactive protein and alpha fetoprotein in HCC patients receiving lenvatinib and pembrolizumab therapy (Wu et al., 2024). The composite measure, termed the CRAFITY score, demonstrated its utility as an independent predictor of overall survival.

In addition to clinical prediction models, other contributions explored molecular biomarkers and signaling pathways associated with HCC progression. Wang et al. developed a six-gene immune activation-related signature that successfully stratified patients according to survival risk and provided mechanistic insights into tumor progression and therapeutic resistance. The study further identified *RORC* as a potential regulator of HCC aggressiveness, with effects mediated by *CDC6* derepression. Similarly, Zhuo et al. established a prognostic model based on immune- and metabolism-related genes, emphasizing the importance of immune-metabolic interactions in HCC biology and their potential clinical relevance.

The role of the tumor microenvironment and immune regulation was also examined in several contributions. Using single-cell RNA sequencing approaches, Li et al. characterized mitophagy-associated cellular subpopulations within the HCC microenvironment and demonstrated their potential contribution to tumor progression through regulation of intercellular communication networks. Complementing these findings, Sun et al. investigated immune cell dynamics following transvascular antitumor interventional therapies and identified specific circulating immune cell populations associated with disease control, highlighting the potential value of immune monitoring in clinical practice (Sun et al.).

This RT further includes studies exploring novel biomarkers with potential diagnostic value. In their systematic review and meta-analysis, Ke et al. investigated the association between circulating adipokines and HCC. The authors reported significant alterations in multiple adipokines and identified visfatin as a promising candidate biomarker, while also highlighting the influence of viral hepatitis on adipokine-associated disease mechanisms. Interestingly, Li et al. identified active visfatin signaling pathways in tumor-associated CD8^+^ T cells, which they hypothesize may lead to increased mitophagy activity and modify cytotoxic T cell function.

**FIGURE 1:**
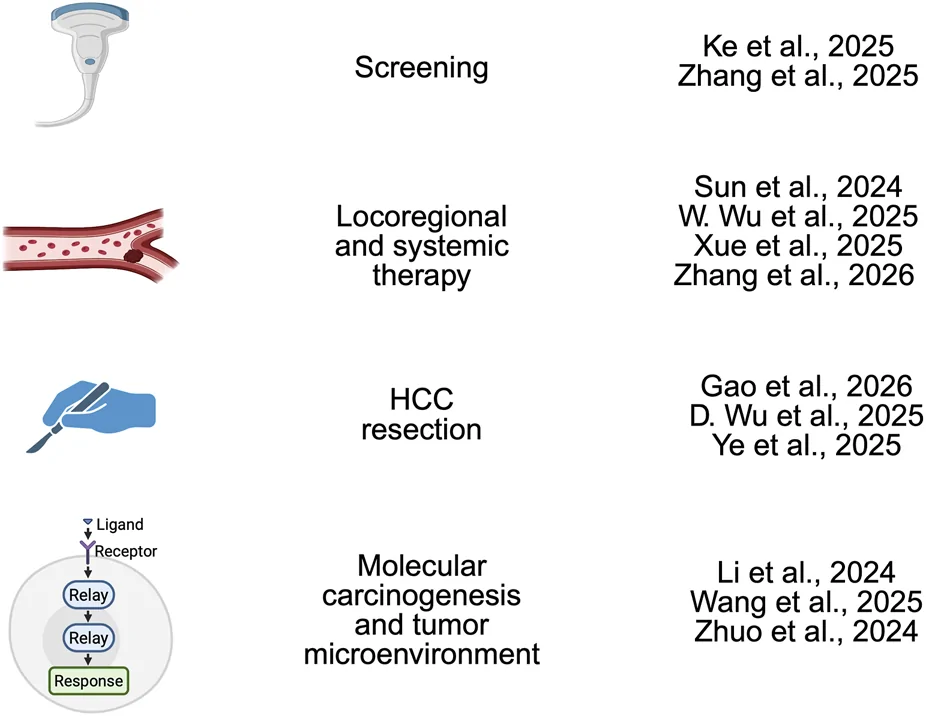
Caption: Domains of hepatocellular carcinoma care impacted by Research Topic contributors. Image created in BioRender web application (BioRender, Toronto, Ontario).

To conclude, this RT brings together recent advances spanning multiple dimensions of HCC research, including therapeutic innovation, prognostic and diagnostic biomarker development, tumor microenvironment regulation, and molecular mechanisms of disease progression (Figure 1). Collectively, these studies provide valuable insights into the biological complexity of HCC and may contribute to the development of more precise diagnostic and therapeutic strategies for affected patients. We, the Research Topic editors, would like to thank and commemorate the participants in these studies, their families and caregivers, and all authors and collaborators for their contributions to science.
