# Correction: Editorial: Hepatocellular carcinoma: from bench to bedside

**DOI:** 10.3389/fgene.2026.1970828

**Published:** 2026-09-01

**Authors:** Evin İşcan, Aaron B. Koenig, Xiaogang Wu

**Affiliations:** 1 Izmir Biomedicine and Genome Center (IBG), Dokuz Eylul University Health Campus, Izmir, Türkiye; 2 Izmir International Biomedicine and Genome Institute (IBG-Izmir), Dokuz Eylul University, Izmir, Türkiye; 3 Celia Scott Weatherhead School of Public Health and Tropical Medicine, Tulane University, New Orleans, LA, United States; 4 The Global NASH/MASH Council, Washington, DC, United States; 5 The University of Texas MD Anderson Cancer Center, Houston, TX, United States

**Keywords:** HCC immune landscape, HCC prognosis, HCC screening, HCC therapy, hepatitis B-related hepatocellular carcinoma, hepatocellular carcinoma, neoadjuvant chemotherapy, tumor microenvironment-TME

Wu et al. (https://doi.org/10.3389/fonc.2025.1504917) was not cited in the article. The citation has now been inserted in paragraph #2 and should read:

“Several contributions focused on improving therapeutic approaches for HCC. In their original article, Zhang et al. compared transarterial chemoembolization combined with donafenib (TACE + D) and transarterial chemoembolization combined with donafenib and camrelizumab (TACE + D + C) in patients with unresectable HCC. The authors demonstrated that the addition of camrelizumab significantly improved treatment responses and survival outcomes while maintaining a manageable safety profile. Similarly, Xue et al. evaluated the efficacy of transarterial chemoembolization combined with lenvatinib and PD-1 blockade in patients with intermediate-stage HCC exceeding the up-to-7 criteria. Their findings showed improved progression-free survival, particularly in progression to macrovascular invasion or extrahepatic spread, further supporting the growing role of immunotherapy-based combination strategies in HCC treatment. Complementing these studies, Wu et al. performed a systematic review and meta-analysis evaluating neoadjuvant systemic therapies in resectable HCC. Their results demonstrated encouraging pathological and clinical response rates, particularly for treatment regimens combining targeted agents and immune checkpoint inhibitors (**Wu et al., 2025**).”

Sun et al. (https://doi.org/10.3389/fimmu.2024.1450525) was not cited in the article. The citation has now been inserted in paragraph #5 and should read:

“The role of the tumor microenvironment and immune regulation was also examined in several contributions. Using single-cell RNA sequencing approaches, Li et al. characterized mitophagy-associated cellular subpopulations within the HCC microenvironment and demonstrated their potential contribution to tumor progression through regulation of intercellular communication networks. Complementing these findings, Sun et al. investigated immune cell dynamics following transvascular antitumor interventional therapies and identified specific circulating immune cell populations associated with disease control, highlighting the potential value of immune monitoring in clinical practice (**Sun et al., 2024**).”

The original version of this article has been updated.

